# Association of blood pressure variability with orthostatic intolerance symptoms

**DOI:** 10.1371/journal.pone.0179132

**Published:** 2017-06-07

**Authors:** Jun-Sang Sunwoo, Tae-Won Yang, Do-Yong Kim, Jung-Ah Lim, Tae-Joon Kim, Jung-Ick Byun, Jangsup Moon, Soon-Tae Lee, Keun-Hwa Jung, Kyung-Il Park, Ki-Young Jung, Manho Kim, Sang Kun Lee, Kon Chu

**Affiliations:** 1 Department of Neurology, Soonchunhyang University College of Medicine, Seoul, South Korea; 2 Department of Neurology, Gyeongsang National University Changwon Hospital, Changwon, South Korea; 3 Department of Neurology, Comprehensive Epilepsy Center, Seoul National University Hospital, Seoul, South Korea; 4 Program in Neuroscience, Seoul National University College of Medicine, Seoul, South Korea; 5 Department of Neurology, National Center for Mental Health, An affiliate of the Ministry for Health & Welfare, Seoul, South Korea; 6 Department of Neurology, Kyung Hee University Hospital at Gangdong, Seoul, South Korea; 7 Department of Neurology, Seoul National University Hospital Healthcare System Gangnam Center, Seoul, South Korea; 8 Protein Metabolism Medical Research Center, Seoul National University College of Medicine, Seoul, South Korea; The University of Tokyo, JAPAN

## Abstract

The short-term blood pressure variability (BPV) reflects autonomic regulatory mechanisms. However, the influence of BPV in orthostatic intolerance (OI) is unknown. Herein, we assessed BPV profiles in patients with OI and determined their association with orthostatic symptoms. In this cross-sectional study, we prospectively enrolled 126 patients presenting with OI at the Seoul National University Hospital from December 2014 to August 2016. Among them, those with other neurological diseases (n = 8) and insufficient BP measurements (n = 15) were excluded. The degree of OI symptoms were measured using the self-administered orthostatic intolerance questionnaire (OIQ). All patients underwent ambulatory BP monitoring and we calculated the standard deviation and coefficient of variation as a measure of BPV. The mean age was 48.6 years and the average of the total OIQ score was 11.6. The severe OI group had higher BPV values than the mild group, although mean BP profiles did not differ significantly. Correlation analysis demonstrated that the orthostatic symptoms were positively correlated with diastolic BPV for the total and awake periods. Multiple linear regression analysis revealed that diastolic BPV (*B* = 0.46, p = 0.031) and current smoking (*B* = 4.687, p = 0.018) were independent factors for higher OI symptom scores after adjusting for covariates. The results of the current study demonstrated that a positive correlation exists between BPV and OI symptoms. Further studies are required to confirm the present findings and understand the neural mechanisms contributing to the excessive BPV in patients with OI.

## Introduction

Orthostatic intolerance (OI) refers to symptoms and signs caused by an upright posture, which can be relieved by lying down [1]. Common presentations include dizziness, lightheadedness, blurred vision, headache, and fainting. OI can also manifest as various nonspecific symptoms, such as concentration difficulties, anxiety, chest discomfort, and tremulousness [2]. OI is a common condition in the adult population; prior studies reported the prevalence of orthostatic hypotension (OH) ranging between 14% and 30.3% and orthostatic dizziness ranging between 4.8% and 19.7% [3–5]. OI is a syndrome consisting of different clinical variants, such as OH, postural orthostatic tachycardia syndrome (POTS), and vasovagal syncope [6]. Accumulating evidence has shown that disruption of autonomic hemodynamic regulation contributes to the mechanisms of these OI subtypes with abnormal orthostatic responses [1, 7–9]. However, a considerable number of patients with OI show normal orthostatic vital sign response in clinical practice. This subgroup remains undiagnosed based on the current classification system. Furthermore, little is known about the pathophysiology and treatment of OI without excessive hypotension or tachycardia.

Blood pressure (BP) continuously fluctuates and its variability indicates the complex interaction between multiple cardiovascular mechanisms, which are mediated by central autonomic regulation, sympathetic vascular modulation, baroreflex, and humoral influences [10, 11]. Although there are several types of blood pressure variability (BPV), the short-term BPV with a time range between minutes and hours represents the effects of autonomic and vasomotor modulation [12]. It can be measured noninvasively by ambulatory BP monitoring. Of note, BPV gained attention because of the findings that it acts as an independent risk factor for cardiovascular diseases [13, 14]. Accumulating evidence has shown that increased BPV is associated with target organ damage, including left ventricular hypertrophy and carotid atherosclerosis [15, 16]. Furthermore, BPV is considered to reflect central and reflex autonomic regulation [12]. Although OI was reported to involve abnormal autonomic responses [17], there has been little information regarding the influence of BPV in OI. Therefore, in this study, we evaluated short-term BPV profiles in patients presenting with OI by using ambulatory BP monitoring and determined the association between the degree of BPV and OI symptoms.

## Materials and methods

### Subjects

We prospectively enrolled 126 patients who presented with OI symptoms at the Department of Neurology of Seoul National University Hospital from December 2014 to August 2016. OI was defined as the development of any symptoms of cerebral hypoperfusion or sympathetic activation upon standing upright, such as dizziness, headache, and faintness. All patients were examined by the authors (K.C. and S. K. L.) and underwent ambulatory BP monitoring and orthostatic vital sign measurement, as well as laboratory and radiological investigations to identify potential causes of OI symptoms. Additionally, we investigated the medical conditions and the current medications that could cause or aggravate OI. Consequently, we excluded 8 patients with an alternative diagnosis that could explain their clinical presentations: epilepsy (n = 5); benign paroxysmal positional vertigo (n = 2); paraneoplastic encephalitis (n = 1). We also excluded 15 patients who obtained valid BP measurements of less than 70% within the total recordings. Finally, a total of 103 patients were analyzed. This study was approved by the institutional review board of Seoul National University Hospital (1410-012-615) and was conducted in compliance with the Declaration of Helsinki and the Good Clinical Practice guidelines. The authors obtained written informed consent from all patients before they were enrolled into the study.

### Orthostatic vital sign response

Orthostatic vital sign responses were measured in a supine position and at 0, 1, 3, 5, and 10 min after standing [18]. Considering its fluctuation during the day, all patients underwent this test twice separately in the morning and evening. OH and POTS were defined according to the 2011 consensus criteria [19]. Patients were classified as OH or POTS if they fulfilled the corresponding criteria at least once. It should be noted that patients with OH or POTS were not excluded from the study, because these conditions are considered common subtypes of OI syndrome rather than secondary causes that needed to be excluded [1].

### Ambulatory blood pressure monitoring

Noninvasive ambulatory BP monitoring was performed with the WatchBP O3 device (Microlife, Golden, CO) that automatically recorded BP and heart rate (HR) every 30 min during the daytime (6 a.m.– 10 p.m.) and once an hour during the nighttime (10 p.m.– 6 a.m.). We set the BP measurement frequency as once an hour during the nighttime to minimize sleep disturbance. After the BP monitoring, every patient reported the time when they went to bed and woke up in a diary. Based on these time points, we analyzed the monitoring data separately for the total, awake, and sleep periods. Mean BP, nocturnal BP dipping, and morning BP surge were calculated for systolic and diastolic BP separately. Mean BP was calculated as the sum of BP values divided by the number of recordings during the corresponding time periods. Nocturnal BP dipping was calculated as follows: (awake BP—sleep BP) / sleep BP ×100 (%). Morning BP surge was calculated as the morning BP minus the lowest BP during sleep; morning BP was defined as the average of first three measurements after waking up.

### Blood pressure variability

BPV was calculated from the ambulatory BP monitoring data by using two variability indices: the standard deviation (SD) and the coefficient of variation (CV). Both SD and CV are commonly used time-domain parameters for short-term BPV [12]. CV was calculated as the average SD divided by the corresponding mean BP and multiplied by 100, and represented the normalized measure of BPV. We computed the systolic BPV (SBPV) and diastolic BPV (DBPV) separately for the total, awake, and sleep periods.

### Orthostatic intolerance questionnaire

The OI symptoms were evaluated using the orthostatic intolerance questionnaire (OIQ), which has been previously shown to be valid and reliable [2, 20]. The OIQ was designed to assess orthostatic symptoms over the last month. The questionnaire consisted of 10 items assessing the presence and frequency of each orthostatic symptom: nausea, tremor in hands, dizziness, palpitation, headache, profuse perspiration, blurred vision, chest discomfort, lightheadedness, and concentration difficulties. Each item was scored from 0 to 4, with 0 indicating no symptom and 4 indicating frequent occurrences more than once a day. We used the total OIQ scores by summing up the 10 item scores as an overall measure of the OI symptoms.

### Statistical analysis

Comparisons of clinical characteristics and BP parameters between two groups were performed by the Student’s t-test and Pearson’s chi-square test according to the nature of variables. Differences according to the orthostatic vital sign response were compared using the analysis of variance with the Bonferroni post-hoc test. The correlation between the BPV and OIQ scores was examined by the Pearson’s correlation analysis. A multiple linear regression analysis was performed to determine the independent association between BPV and OIQ scores. Variables with a p-value of less than 0.1 in simple linear regression analysis were included as covariates. Age and mean BP were additionally included to control for the potential confounding effects. A two-tailed p-value < 0.05 was considered statistically significant. All statistical analyses were performed using SPSS version 18 (SPSS Inc., Chicago, IL).

## Results

### Clinical features

**Fig 1:**
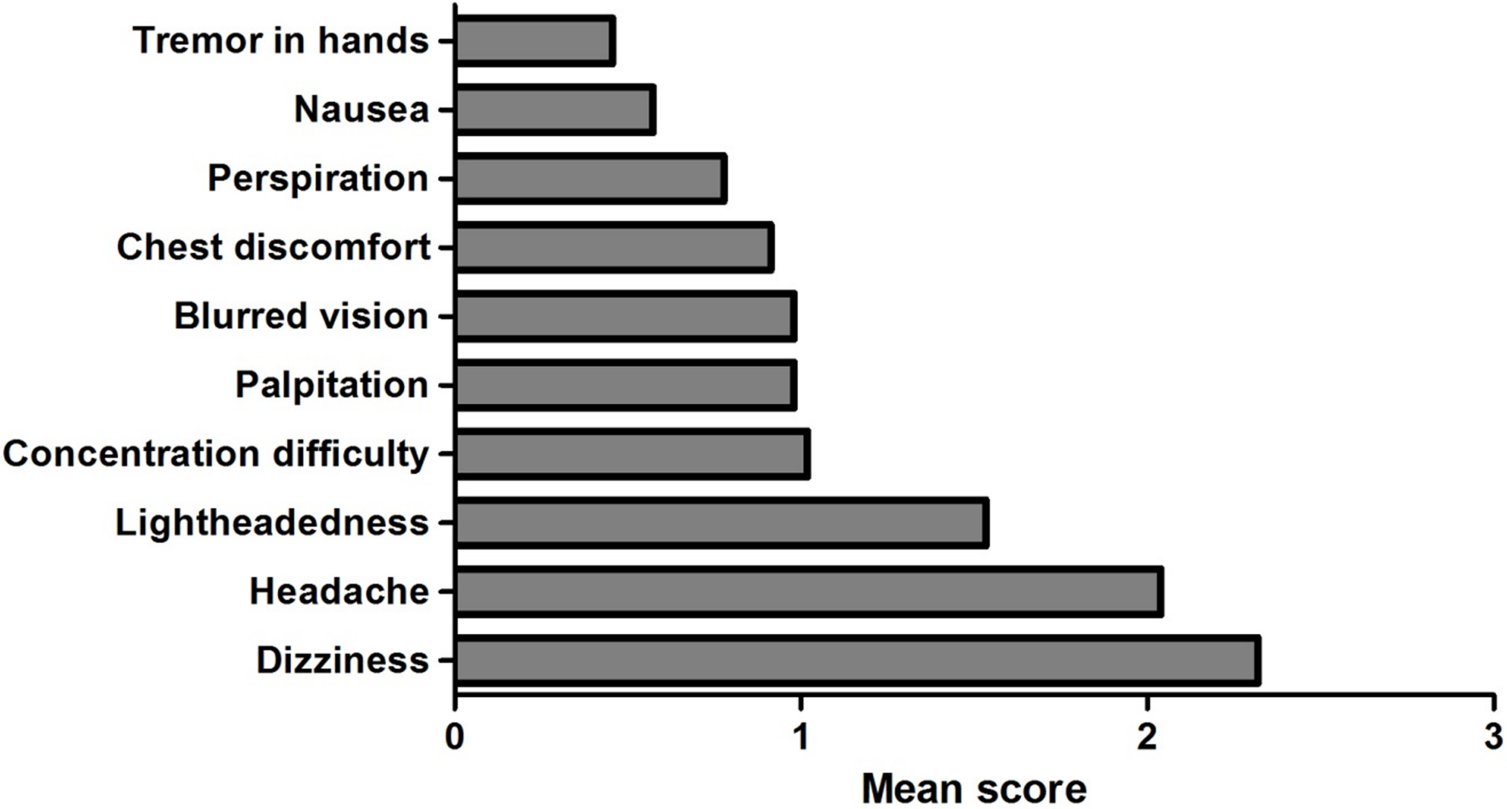
Orthostatic intolerance questionnaire scores for each item in total patients (n = 103). Each item score ranges from 0 to 4 according to the frequency of orthostatic symptoms.

A total of 103 patients were evaluated in this study. Overall, the mean age was 48.6 ± 17.5 years, and 61 (59.2%) of the patients were women. The average of the total OIQ scores was 11.6 ± 6.2. Among 10 items, dizziness, headache, and lightheadedness were the three most common symptoms, which accounted for 50.8% of total scores (Fig 1). The orthostatic vital sign response was normal in 43 (41.7%) patients while 33 (32%) showed OH and 27 (26.2%) showed POTS. However, total OIQ scores did not differ according to the orthostatic responses (normal, 10.7 ± 6.2; OH, 12 ± 5.8; POTS, 12.6 ± 6.8; p = 0.441). Demographic and BP profiles are summarized in Table 1.

**Table 1:** Clinical characteristics and ambulatory blood pressure measurement profiles.

Variables	Total (n = 103)	Mild OI (n = 56)	Severe OI (n = 47)	p-value
Age, yr	48.6 (17.5)	50 (15.5)	47 (19.7)	0.395
Sex, female (%)	61 (59.2)	31 (55.4)	30 (63.8)	0.383
BMI, kg/m^2^	23.9 (3.5)	24.1 (3.3)	23.6 (3.7)	0.475
Current smoking (%)	11 (10.7)	4 (7.1)	7 (14.9)	0.205
Total OIQ score	11.6 (6.2)	6.9 (2.7)	17.2 (4.3)	< 0.001
Valid reading, %	83.9 (8.4)	84.6 (8.9)	83.2 (7.8)	0.395
Orthostatic response				0.176
Normal (%)	43 (41.7)	28 (50)	15 (31.9)	
Hypotension (%)	33 (32.0)	15 (26.8)	18 (38.3)	
Tachycardia (%)	27 (26.2)	13 (23.2)	14 (29.8)	
Medical history				
Hypertension (%)	18 (17.5)	9 (16.1)	9 (19.1)	0.682
Diabetes (%)	5 (4.9)	2 (3.6)	3 (6.4)	0.658
CAD (%)	6 (5.8)	3 (5.4)	3 (6.4)	1
Hyperlipidemia (%)	10 (9.7)	4 (7.1)	6 (12.8)	0.506
Medication (%)*	30 (29.1)	14 (25)	16 (34)	0.314
Blood tests				
Total Cholesterol, mg/dL	177.1 (32.4)	177.8 (30.9)	176.2 (34.4)	0.805
BUN, mg/dL	13.6 (3.8)	14 (3.6)	13.1 (4)	0.206
Sodium, mmol/L	140.4 (1.9)	140.7 (1.7)	140 (2)	0.073
BP monitoring				
SBP, mmHg				
Total	114.1 (12.4)	115.5 (14)	112.4 (10.1)	0.198
Awake	115.6 (12.5)	116.7 (14)	114.2 (10.4)	0.310
Sleep	111.1 (13.7)	113 (14.8)	108.8 (11.9)	0.122
DBP, mmHg				
Total	69.7 (7.6)	70.5 (7.9)	68.6 (7.2)	0.196
Awake	71 (7.6)	71.7 (8.1)	70.1 (6.9)	0.263
Sleep	67.2 (8.8)	68.4 (8.4)	65.9 (9.1)	0.156
Mean HR, bpm				
Total	65.4 (8.9)	65 (8.2)	65.9 (9.7)	0.585
Awake	67.2 (10.7)	66.1 (11.4)	66.5 (9.8)	0.277
Sleep	61.1 (10.1)	60.9 (9.2)	61.3 (11.1)	0.838

Data are presented as mean (standard deviation) or number (percentage). Comparisons between two groups were performed by the student’s t-test or chi-square test. Abbreviations: OI, orthostatic intolerance; BMI, body mass index; OIQ, orthostatic intolerance questionnaire; CAD, coronary artery disease; BUN, blood urea nitrogen; SBP, systolic blood pressure; DBP, diastolic blood pressure; HR, heart rate.

*Medication which can cause or aggravate orthostatic intolerance including antihypertensive, vasodilator drugs, and alpha blockers

We classified the patients into two subgroups based on the total OIQ score as follows: mild OI group (total OIQ score ≤ 11; n = 56) versus severe OI group (total OIQ score ≥ 12; n = 47). As shown in Table 1, clinical characteristics including age, sex, body mass index (BMI), and the prevalence of medication use did not differ between the two groups. Furthermore, there were no significant differences in the mean BP levels during the total, awake, and sleep periods. The mean HR of the total patients was 65.4 ± 8.9 bpm and did not significantly differ between the mild and severe symptom groups (p = 0.585). In addition, orthostatic vital sign responses were similar between the two groups (p = 0.176). There were no differences in results of blood tests including hemoglobin, hematocrit, total cholesterol, blood urea nitrogen, creatinine, sodium, and thyroid function test.

### Blood pressure variability

**Fig 2:**
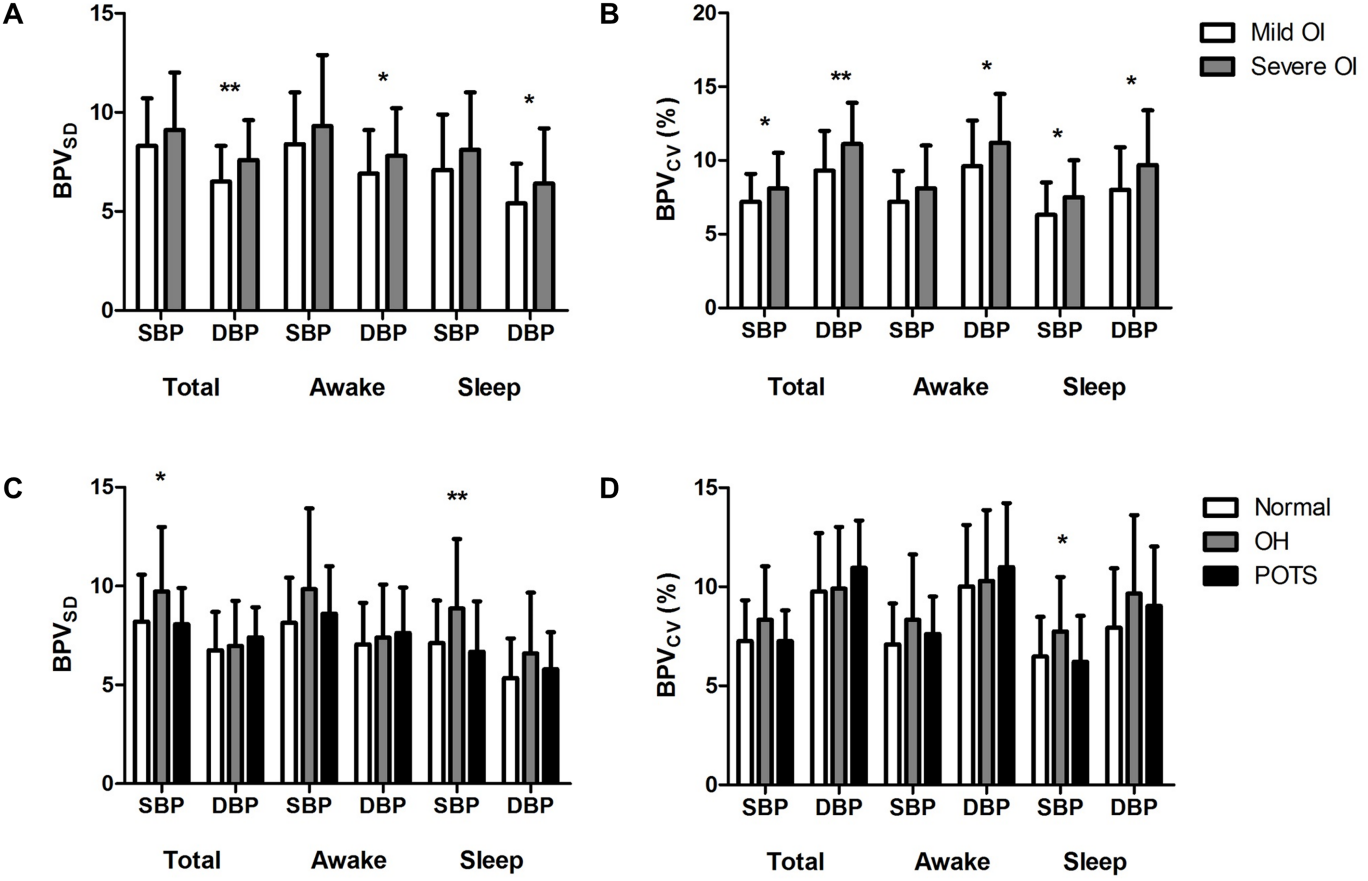
Blood pressure variability according to symptom severity and orthostatic vital signs. (A) Blood pressure variability (BPV) measured by the standard deviation (BPV_SD_ [mmHg]) between patients with mild (n = 56) and severe (n = 47) orthostatic intolerance symptoms. (B) BPV measured by the coefficient of variation (BPV_CV_ [%]) between the mild and severe symptom groups. (C, D) BPV differences according to orthostatic vital sign responses: OH (n = 33), POTS (n = 27), and normal (n = 43). (C) BPV_SD_ (mmHg) among the three groups. Post-hoc test: total systolic BPV_SD_ (OH vs. normal, p = 0.038; OH vs. POTS, p = 0.045), sleep systolic BPV_SD_ (OH vs. normal, p = 0.021; OH vs. POTS, p = 0.008). (D) BPV_CV_ (%) among OH, POTS, and normal groups. Post-hoc test: sleep systolic BPV_CV_ (OH vs. normal, p = 0.067; OH vs. POTS, p = 0.04). Abbreviations: SBP, systolic blood pressure; DBP, diastolic blood pressure; OI, orthostatic intolerance; OH, orthostatic hypotension; POTS, postural orthostatic tachycardia syndrome. * p < 0.05, ** p < 0.01. Error bars indicate the standard deviation.

We measured both BPV_SD_ and BPV_CV_ for total, awake, and sleep periods. When comparing BPV between two groups, severe OI group had significantly increased levels of BPV. The severe OI group had a higher DBPV_SD_ than the mild OI group during total (7.6 ± 2 vs. 6.5 ± 1.8 mmHg, p = 0.007), awake (7.8 ± 2.4 vs. 6.9 ± 2.2 mmHg, p = 0.033), and sleep (6.4 ± 2.8 vs. 5.4 ± 2 mmHg, p = 0.042) periods (Fig 2A). However, SBPV_SD_ were not different between the two groups. When comparing the two groups by BPV_CV_, there were differences in both SBPV and DBPV (Fig 2B). SBPVcv of the severe OI group were significantly higher than those of the mild group during the total (8.1 ± 2.4 vs. 7.1 ± 1.9%, p = 0.042) and sleep (7.5 ± 2.5 vs. 6.3 ± 2.2%, p = 0.01) period recordings. DBPV_CV_ was also increased in the severe OI group during the total (11.1 ± 2.8 vs. 9.3 ± 2.7%, p = 0.002), awake (11.2 ± 3.3 vs. 9.6 ± 3.1%, p = 0.014), and sleep (9.7 ± 3.7 vs. 8 ± 2.9%, p = 0.01) period recordings. In summary, the patients with severe OI had significantly increased BPV compared to those with mild OI, and DBPV demonstrated more consistent results than SBPV.

Next, we compared BPV profiles according to orthostatic vital sign responses. Patients with OH had higher SBPV_SD_ compared to other groups during the total (9.7 ± 3.3 mmHg for OH, 8.1 ± 1.8 mmHg for POTS, 8.2 ± 2.4 mmHg for normal, p = 0.018) and sleep (8.9 ± 3.5 mmHg for OH, 6.7 ± 2.5 mmHg for POTS, 7.1 ± 2.1 mmHg for normal, p = 0.005) periods (Fig 2C). SBPV_CV_ for the sleep period was also higher among OH patients (7.7 ± 2.7% for OH, 6.2 ± 2.3% for POTS, 6.5 ± 2.0% for normal, p = 0.023), but the group differences were less prominent than those of SBPV_SD_ (Fig 2D). However, the DBPV values did not differ significantly among the three groups. Moreover, there were no differences in any BPV parameters between the patients with POTS and those with normal orthostatic responses.

### Correlation analysis

**Fig 3:**
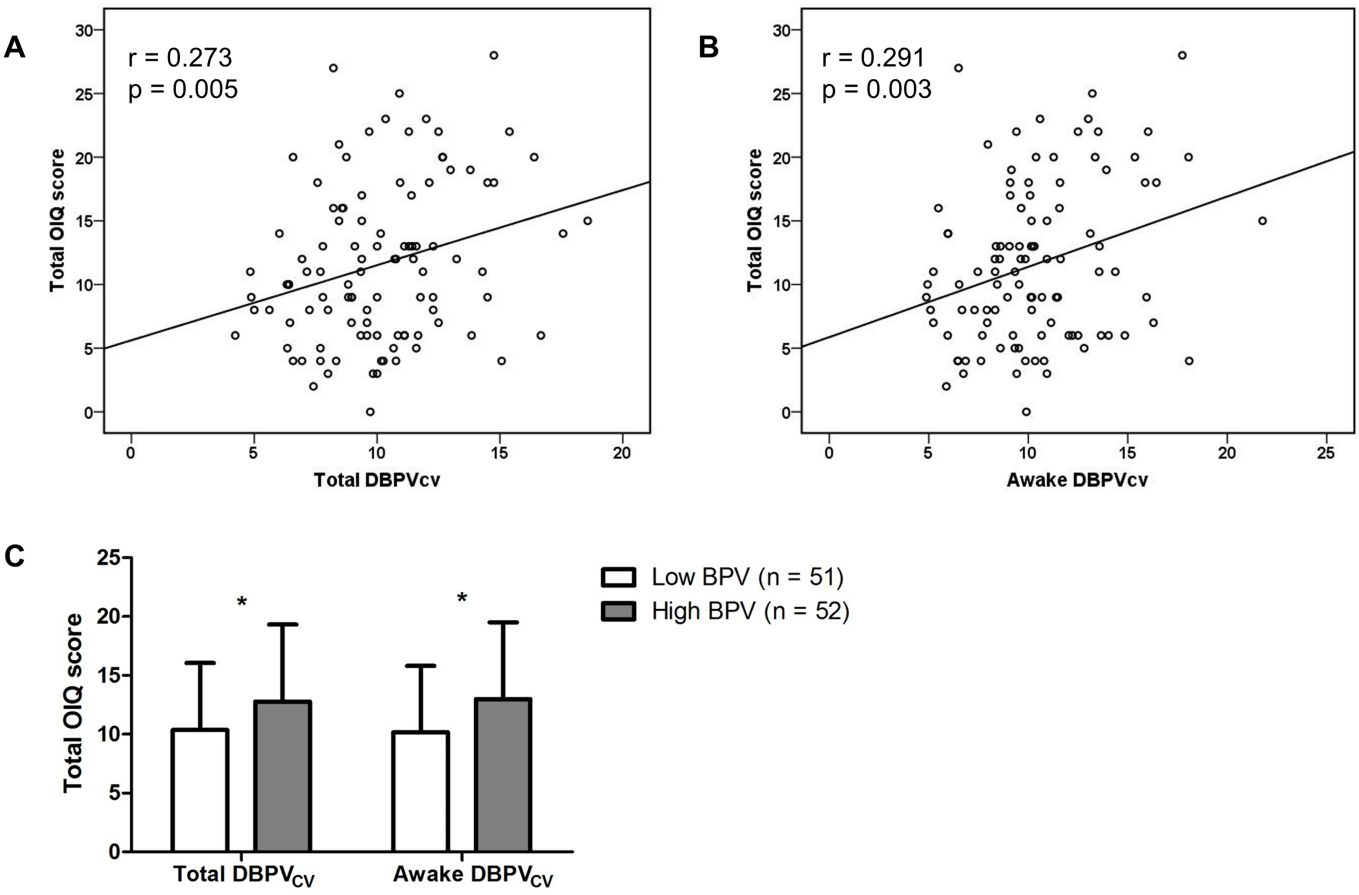
Scatter plots between blood pressure variability and total orthostatic intolerance questionnaire (OIQ) scores (n = 103). (A) Diastolic blood pressure variability measured by the coefficient of variation (DBPV_CV_ [%]) for total recordings. (B) DBPV_CV_ (%) during the awake period. (C) Comparison of total OIQ scores between the high (n = 52) and low (n = 51) BPV groups. The cut-off value of total DBPV_CV_ was 10%, and that of awake DBPV_CV_ was 10.1%. * p < 0.05. Error bars indicate the standard deviation.

Correlation analysis demonstrated that the total orthostatic symptom scores were positively correlated with DBPV for total and awake periods (Table 2; Fig 3A and 3B). Between two indices of DBPV, DBPV_CV_ showed the higher level of linear correlation with the total OIQ scores than did DBPV_SD_ in both total and awake recordings. However, any SBPV values were not significantly related with the orthostatic symptoms. Further, we evaluated the correlation of DBPV_CV_ levels with each orthostatic symptom score. Among 10 items, dizziness, the most common orthostatic symptom, had the strongest correlation with the DBPVcv measured during the total (r = 0.26, p = 0.008) and awake (r = 0.285, p = 0.004) periods. Other symptoms significantly associated with the DBPV_CV_ value included headache, blurred vision, and chest discomfort (S1 Table). Next, we divided the patients into the high and low BPV groups based on the DBPV_CV_ value and compared the difference in the total OIQ scores between the two groups. As expected, the high BPV group had a significantly higher OIQ score than the low BPV group (total DBPV_CV_, 12.8 ± 6.5 vs. 10.4 ± 5.7%, p = 0.048; awake DBPV_CV_, 13 ± 6.5 vs. 10.2 ± 5.6%, p = 0.022; Fig 3C).

**Table 2:** Correlation between blood pressure variability and orthostatic intolerance symptom scores.

Variables	SD†		CV‡	
	r	p-value	r	p-value
Total SBPV	0.117	0.239	0.157	0.114
Total DBPV	0.22	0.025*	0.273	0.005**
Awake SBPV	0.136	0.171	-0.015	0.88
Awake DBPV	0.244	0.013*	0.291	0.003**
Sleep SBPV	0.105	0.289	0.169	0.088
Sleep DBPV	0.1	0.317	0.158	0.111

The correlation coefficient (r) was estimated by the Pearson’s correlation analysis (n = 103). Abbreviations: SD, standard deviation; CV, coefficient of variation; SBPV, systolic blood pressure variability; DBPV, diastolic blood pressure variability

*p < 0.05

**p < 0.01

^†^Blood pressure variability measured by the standard deviation,

^‡^Blood pressure variability measured by the coefficient of variation

Simple linear regression analyses demonstrated that female sex (*B* = 2.447, p = 0.049) and current smoking (*B* = 4.121, p = 0.037) as well as BPV values had a significant positive correlation with the total OIQ scores (S2 Table). Among laboratory test results, serum blood urea nitrogen (BUN; *B* = -0.458, p = 0.004) and sodium (*B* = -0.723, p = 0.025) levels were negatively correlated with the OIQ scores. As opposed to BPV, mean BP and HR levels were not associated with OI symptoms. The presence of excessive hypotension or tachycardia during the orthostatic test also had no influence on the orthostatic symptoms. Furthermore, morning BP surge or nocturnal BP dipping had no significant relationship with OI. Subsequently, multiple linear regression analyses were performed to determine the independent association between BPV and the OI symptom scores. We used two separate models according to the time period: the total period for model 1 and the awake period for model 2. Sex, BMI, current smoking, as well as serum BUN and sodium levels, which had p-value < 0.1 in the univariate analyses, were entered into the models as covariates. Additionally, age and the corresponding mean BP levels were included for adjustment. Multiple regression analyses demonstrated that DBPV_CV_ remained an independent factor for the OI symptoms after adjusting for the covariates in both models (*B* = 0.46, p = 0.031 in the model 1; *B* = 0.429, p = 0.02 in the model 2; Table 3). Notably, current smoking was also shown to significantly affect the total OIQ scores. Furthermore, the multiple linear regression models for the specific item score showed that DBPV_CV_ had an independent linear relationship with orthostatic dizziness (*B* = 0.125, p = 0.008 in the model 1; *B* = 0.115, p = 0.004 in the model 2; S3 Table). In these models, age and current smoking had a positive correlation and BMI had a negative correlation with the dizziness score.

**Table 3:** Multiple linear regression analyses for orthostatic intolerance symptom scores.

Variables	*B*	95% CI	p-value	R^2^
Model 1				0.234
Age, yr	0.045	-0.037–0.127	0.28	
Sex, female	2.127	-0.311–4.566	0.086	
Body mass index, kg/m^2^	-0.232	-0.605–0.142	0.221	
Current smoking	4.687	0.821–8.554	0.018	
Serum BUN, mg/dL	-0.340	-0.687–0.007	0.054	
Serum Sodium, mmol/L	-0.485	-1.082–0.113	0.11	
Mean total DBP, mmHg	-0.006	-0.188–0.177	0.952	
Total DBPV_CV_, %	0.460	0.042–0.877	0.031	
Constant	81.601	-3.557–166.76	0.06	
Model 2				0.24
Age, yr	0.43	-0.038–0.124	0.29	
Sex, female	2.099	-0.324–4.522	0.089	
Body mass index, kg/m^2^	-0.235	-0.597–0.126	0.199	
Current smoking	4.384	0.537–8.231	0.026	
Serum BUN, mg/dL	0.324	-0.67–0.022	0.066	
Serum Sodium, mmol/L	-0.522	-1.116–0.072	0.084	
Mean awake DBP, mmHg	-0.016	-0.189–0.158	0.857	
Awake DBPV_CV_, %	0.429	0.07–0.787	0.02	
Constant	87.715	2.892–172.54	0.043	

The total score of orthostatic intolerance questionnaires is a dependent variable (n = 103). *B* denotes the unstandardized coefficient. Model 1, F(8, 94) = 3.589, p = 0.001, blood pressure and variability variables derived from the total recordings; Model 2, F(8, 94) = 3.719, p = 0.001, blood pressure and variability variables derived from the awake recordings. Abbreviations: CI, confidence interval; BUN, blood urea nitrogen; DBP, diastolic blood pressure; DBPV_CV_, diastolic blood pressure variability measured by the coefficient of variation.

## Discussion

The results of the present study demonstrated that a positive correlation exists between BPV and OI symptoms. Although this is the first study to show the influence of BPV in patients with OI, accumulating evidence suggests that increased BPV is implicated in vasovagal syncope, which is considered a specific variant of OI syndrome [1, 21]. The cardiovascular time series analysis suggested that patients with vasovagal syncope had significantly increased BPV during orthostatic challenge compared to controls [22]. Excessive BPV was accompanied by sympathetic hyperactivity and vagal withdrawal, which suggested autonomic dysregulation in response to orthostatic stress. Similarly, an ambulatory BP monitoring study reported the increase of BPV in patients with neurally mediated reflex syncope, regardless of the head-up tilt results [23]. In this regard, our findings extended the concept that increased BPV is associated with the altered autonomic regulation from classical vasovagal syncope to OI without pathological orthostatic responses. It is suggested that excessive BPV might contribute to OI symptoms in those who had normal orthostatic vital signs. Further studies with a large sample size and neurophysiological evidence of altered BPV will be needed to confirm the hypothesis. Between the two BPV indices, BPV_CV_ has the advantage of being less affected by the changes of mean BP levels [12]. In this aspect, BPV_CV_ might better correlate with the OI symptom scores than BPV_SD_. However, why OI symptoms were independently associated with DBPV rather than SBPV is difficult to explain. Further study is required to elucidate its clinical relevance.

Concerning the neural mechanisms of excessive BPV, it has been established that the sympathetic drive substantially contributes to BPV [24, 25]. A previous study showed that atropine-induced parasympathetic inhibition significantly reduced HR variability, but did not affect BPV [26]. In contrast, renal sympathetic denervation, which reduces both renal and central sympathetic activity, significantly decreased BPV in patients with refractory arterial hypertension [27]. In addition, direct measurements of efferent postganglionic muscle sympathetic nerve activity showed a positive interaction with BPV values [28]. It is also noteworthy that the sympathetic activity affected the daytime BPV but not the nighttime BPV in the above mentioned studies. In line with this, our data showed that BPV is significantly associated with OI symptoms during wakefulness rather than during sleep. The diurnal BPV difference might be attributed to sleep itself, which physiologically lowers sympathetic nerve activity, mean BP level, and BPV [11, 29]. Another possible explanation is that sympathetic mechanisms predominantly mediate the cardiovascular modulation sensitive to environmental and behavioral factors which are eliminated during sleep [30]. Taken together, considering that sympathetic activity serves as the key regulator of BPV, it is presumed that sympathetic hyperactivity might underlie the disease mechanism of OI. In addition, BPV measurement through ambulatory BP monitoring could be used to objectively monitor the symptom progress and treatment response in OI. Further research is warranted to gather direct evidence of this hypothesis and to determine the beneficial effects of therapeutics to counterbalance sympathetic overactivity in OI [31].

Along with BPV, cigarette smoking was found to be an independent factor for the OI symptoms in this study. As for OH, current smoking was found to be an independent determinant for the impairment of orthostatic reaction [32]. A sympathetic microneurography study in hypertensive patients demonstrated that smoking was associated with chronic sympathetic activation [33]. Smoking was reported to induce sympathetic activation by peripheral adrenergic stimulation and partial impairment of baroreflex [34]. Therefore, smoking might be responsible for orthostatic impairment by depleting the hemodynamic sympathetic reserve. In addition, low BMI was independently associated with orthostatic dizziness in our patients. Similarly, patients with OH and POTS with low blood flow were shown to have lower BMI compared to controls [32]. The inverse correlation between BMI and plasma angiotensin II levels in POTS suggested enhanced sympathetic tone in those with low BMI [35, 36]. Taken together, these finding suggested that low BMI reflected the hypovolemic status of the patients and that compensatory sympathetic activation might contribute to the development of OI [37].

There are several limitations in this study. Because our study was limited to patients with OI, we do not know whether there is a significant difference in BPV between patients with OI and healthy controls. If this is true, it would substantiate the hypothesis that increased BPV contributes to the development of OI. Moreover, the heterogeneity of the study subjects is another limitation, because the mechanism of excessive BPV might be different among OI subtypes. Further, the OIQ mainly focuses on the presence and frequency of OI symptoms, and does not exactly represent the severity of OI symptoms. In addition, we did not estimate the degree of physical activity or posture during ambulatory BP monitoring, which might have influenced the BPV profile of the subjects. Relatively low BP recording frequency and low percentages of valid reading were other limitations. Therefore, we cannot rule out the possibility that a low frequency of nighttime BP measurements might have affected the sleep BPV data.

## Conclusions

The results of this study showed that excessive BPV was an independent factor of severe orthostatic symptoms in patients with OI. Of particular importance is that the association of BPV was not limited to the classical OI subtypes, but also involved OI with normal orthostatic responses. Our findings provide some insight into the hypothesis that abnormally increased BPV might contribute to OI. As an objective measure of OI symptoms, BPV evaluation might be used to observe the disease course or treatment response in patients with OI. Further studies are warranted to validate the present findings and understand the neural mechanisms underlying the abnormal enhancement of BPV in OI.

## Supporting information

S1 Table Correlation of blood pressure variability with specific orthostatic intolerance symptoms. (DOC)

S2 Table Simple linear regression analyses for total orthostatic intolerance questionnaire scores. (DOC)

S3 Table Multiple linear regression analyses for orthostatic dizziness scores. (DOC)
