# Supplementary material for: Association of blood pressure variability with orthostatic intolerance symptoms
Source: PLoS One. 2017 Jun 7;12(6):e0179132. doi: 10.1371/journal.pone.0179132 (PMC5462414; doi:10.1371/journal.pone.0179132)
Supplement: S1 Table — (DOC) [file pone.0179132.s001.doc]

**S1 Table. Correlation of blood pressure variability with specific orthostatic intolerance symptoms.**

| Items | Total DBPV_CV_ | | Awake DBPV_CV_ | | Total DBPV_SD_ | | Awake DBPV_SD_ | |
| --- | --- | --- | --- | --- | --- | --- | --- | --- |
|  | r | p-value | r | p-value | r | p-value | r | p-value |
| Nausea | 0.112 | 0.26 | 0.037 | 0.712 | 0.13 | 0.19 | 0.056 | 0.571 |
| Tremor in hands | 0.165 | 0.097 | 0.147 | 0.137 | 0.158 | 0.111 | 0.143 | 0.149 |
| **Dizziness** | 0.26** | 0.008 | 0.285** | 0.004 | 0.208* | 0.035 | 0.245* | 0.013 |
| Palpitation | 0.072 | 0.469 | 0.165 | 0.095 | 0.073 | 0.462 | 0.17 | 0.086 |
| **Headache** | 0.118 | 0.233 | 0.243* | 0.013 | 0.102 | 0.307 | 0.226* | 0.022 |
| Profuse perspiration | 0.182 | 0.066 | 0.095 | 0.339 | 0.15 | 0.131 | 0.075 | 0.451 |
| **Blurred vision** | 0.204* | 0.039 | 0.211* | 0.033 | 0.113 | 0.257 | 0.124 | 0.213 |
| **Chest discomfort** | 0.218* | 0.027 | 0.179 | 0.071 | 0.241* | 0.014 | 0.195* | 0.048 |
| Lightheadedness | 0.064 | 0.519 | 0.079 | 0.426 | 0.017 | 0.867 | 0.033 | 0.741 |
| Concentration difficulties | 0.072 | 0.473 | 0.067 | 0.498 | 0.02 | 0.84 | 0.019 | 0.181 |

The correlation coefficient (r) was estimated by the Pearson’s correlation analysis. Abbreviations: DBPV, diastolic blood pressure variability; CV, correlation coefficient; SD, standard deviation. n = 103; *p < 0.05, **p < 0.01
