# Supplementary material for: Association of blood pressure variability with orthostatic intolerance symptoms
Source: PLoS One. 2017 Jun 7;12(6):e0179132. doi: 10.1371/journal.pone.0179132 (PMC5462414; doi:10.1371/journal.pone.0179132)
Supplement: S2 Table — (DOC) [file pone.0179132.s002.doc]

**S2 Table. Simple linear regression analyses for total orthostatic intolerance questionnaire scores.**

| Variables | *B* | 95% CI | p-value |
| --- | --- | --- | --- |
| Age, yr | -0.058 | -0.127 - 0.011 | 0.101 |
| Sex, female | 2.447 | 0.006 - 4.888 | 0.049* |
| Body mass index, kg/m^2^ | -0.327 | -0.675 - 0.02 | 0.065 |
| Current smoking | 4.121 | 0.246 - 7.885 | 0.037* |
| Orthostatic vital sign response |  |  |  |
| Hypotension | 0.555 | -2.063 - 3.174 | 0.675 |
| Tachycardia | 1.306 | -1.463 - 4.074 | 0.352 |
| Medical history |  |  |  |
| Hypertension | -0.179 | -3.399 - 3.041 | 0.912 |
| Diabetes | 1.69 | -3.99 - 7.37 | 0.556 |
| Cardiovascular disease | -1.337 | -6.551 - 3.877 | 0.612 |
| Hyperlipidemia | -0.988 | -5.114 - 3.137 | 0.636 |
| Medication use† | 0.669 | -2.019 - 3.358 | 0.622 |
| Blood tests |  |  |  |
| Hemoglobin, g/dL | -0.128 | -1.03 - 0.774 | 0.779 |
| Hematocrit, % | -0.111 | -0.438 - 0.216 | 0.503 |
| Total cholesterol, mg/dL | -0.011 | -0.049 - 0.027 | 0.572 |
| BUN, mg/dL | -0.458 | -0.769 - -0.147 | 0.004** |
| Creatinine, mg/dL | -1.452 | -9.011 - 6.106 | 0.704 |
| Sodium, mmol/L | -0.723 | -1.351 - -0.094 | 0.025* |
| fT4, ng/dL‡ | -1.583 | -5.996 - 2.829 | 0.478 |
| TSH, ng/dL‡ | 0.507 | -1.015 - 2.03 | 0.51 |
| Blood pressure monitoring |  |  |  |
| Successful reading, % | -0.097 | -0.243 - 0.048 | 0.187 |
| No. of valid reading | 0.054 | -0.266 - 0.375 | 0.738 |
| Total period |  |  |  |
| Mean DBP, mmHg | -0.125 | -0.284 - 0.035 | 0.124 |
| DBPV_SD_, mmHg | 0.698 | 0.088 - 1.308 | 0.025* |
| DBPV_CV_, % | 0.59 | 0.18 - 0.999 | 0.005** |
| Mean HR, bpm | -0.002 | -0.14 – 0.136 | 0.976 |
| Awake period |  |  |  |
| Mean DBP, mmHg | -0.112 | -0.272 - 0.048 | 0.168 |
| DBPV_SD_, mmHg | 0.649 | 0.141 - 1.157 | 0.013* |
| DBPV_CV_, % | 0.552 | 0.194 - 0.091 | 0.003** |
| Mean HR, bpm | 0.055 | -0.059 – 0.169 | 0.345 |
| Morning SBP Surge, mmHg | 0.098 | -0.029 - 0.224 | 0.128 |
| Morning DBP Surge, mmHg | 0.025 | -0.125 - 0.175 | 0.739 |
| Nocturnal SBP Dip, % | 0.1 | -0.115 - 0.315 | 0.358 |
| Nocturnal DBP Dip, % | 0.097 | -0.082 - 0.277 | 0.284 |

The total score of orthostatic intolerance questionnaires is a dependent variable (n = 103). *B* denotes the unstandardized coefficient. Abbreviations: CI, confidence interval; BUN, blood urea nitrogen; fT4, free thyroxine; TSH, thyroid stimulating hormone; SBP, systolic blood pressure; DBP, diastolic blood pressure; DBPV_CV_, diastolic blood pressure variability measured by the coefficient of variation; DBPV_SD_, diastolic blood pressure variability measured by the standard deviation; HR, heart rate.

*p < 0.05, **p < 0.01, †Medication which can cause or aggravate orthostatic intolerance including antihypertensive, vasodilator drugs, and alpha blockers, ‡data available in 99 patients.
