# Supplementary material for: Association of blood pressure variability with orthostatic intolerance symptoms
Source: PLoS One. 2017 Jun 7;12(6):e0179132. doi: 10.1371/journal.pone.0179132 (PMC5462414; doi:10.1371/journal.pone.0179132)
Supplement: S3 Table — (DOC) [file pone.0179132.s003.doc]

**S3 Table. Multiple linear regression analyses for orthostatic dizziness scores.**

| Variables | *B* | 95% CI | p-value | R^2^ |
| --- | --- | --- | --- | --- |
| Model 1 |  |  |  | 0.229 |
| Age, yr | 0.02 | 0.003– 0.038 | 0.025 |  |
| Sex, female | 0.065 | -0.469 – 0.599 | 0.810 |  |
| Body mass index, kg/m^2^ | -0.105 | -0.187 - -0.024 | 0.012 |  |
| Current smoking | 1.041 | 0.194 – 1.887 | 0.017 |  |
| Serum BUN, mg/dL | -0.074 | -0.150 – 0.002 | 0.056 |  |
| Serum Sodium, mmol/L | -0.042 | -0.173 – 0.089 | 0.528 |  |
| Mean total DBP, mmHg | -0.002 | -0.042 – 0.038 | 0.91 |  |
| Total DBPV_CV_, % | 0.125 | 0.033 – 0.216 | 0.008 |  |
| Constant | 9.444 | -9.203 – 28.091 | 0.317 |  |
| Model 2 |  |  |  | 0.238 |
| Age, yr | 0.02 | 0.003 – 0.038 | 0.026 |  |
| Sex, female | 0.054 | -0.476 – 0.584 | 0.84 |  |
| Body mass index, kg/m^2^ | -0.106 | -0.185 - -0.027 | 0.009 |  |
| Current smoking | 0.961 | 0.120 – 1.802 | 0.026 |  |
| Serum BUN, mg/dL | -0.07 | -0.146 – 0.006 | 0.07 |  |
| Serum Sodium, mmol/L | -0.052 | -0.182 – 0.078 | 0.426 |  |
| Mean awake DBP, mmHg | -0.006 | -0.044 – 0.0.032 | 0.748 |  |
| Awake DBPV_CV_, % | 0.115 | 0.037 – 0.194 | 0.004 |  |
| Constant | 11.261 | -7.283 – 29.806 | 0.231 |  |

The item score for orthostatic dizziness is a dependent variable (n = 103). *B* denotes the unstandardized coefficient. Model 1, F(8, 94) = 3.449, p = 0.001, blood pressure and variability variables derived from the total recordings; Model 2, F(8, 94) = 3.677, p = 0.001, blood pressure and variability variables derived from the awake recordings. Abbreviations: CI, confidence interval; BUN, blood urea nitrogen; DBP, diastolic blood pressure; DBPV_CV_, diastolic blood pressure variability measured by the coefficient of variation.
